# Multiple Laser Doppler Flowmetry Probes Increase the Reproducibility of Skin Blood Flow Measurements

**DOI:** 10.3389/fphys.2022.876633

**Published:** 2022-05-30

**Authors:** J. Carter Luck, Allen R. Kunselman, Michael D. Herr, Cheryl A. Blaha, Lawrence I. Sinoway, Jian Cui

**Affiliations:** ^1^ Penn State Health Heart and Vascular Institute, Pennsylvania State University College of Medicine, Hershey, PA, United States; ^2^ Department of Public Health Sciences, Pennsylvania State University College of Medicine, Hershey, PA, United States

**Keywords:** skin blood flow, laser Doppler, thermoregulation, reproducibility, microcirculation

## Abstract

Cutaneous microcirculatory perfusion is commonly measured using laser Doppler flowmetry (LDF) probes, which provide a continuous, non-invasive quantification of skin blood flow (SkBF). However, inhomogeneities in the skin’s microvasculature density contribute to a decrease in reproducibility whenever an LDF probe is removed and replaced, as is the case during pre- and post-intervention or between-day measurements. Therefore, this study aimed to determine whether increasing the total number of individual LDF probes in a localized area improves the reproducibility of the measurement. Seven laser Doppler probes were secured in a custom-made acrylic holder designed to attach to the skin’s surface easily. SkBF, local skin temperature (Tsk), and blood pressure (BP) were assessed in 11 participants (6 M, 5 F, 42 ± 15 years). SkBF and Tsk were measured from the dorsal forearm (arm trial) for 5 min. Next, the multi-laser device was moved to the lateral side of the calf (leg trial), and measurements were obtained for 5 min. Each arm and leg trial was cyclically repeated three times, and all trials were separated by intermissions lasting 10–15 min. The average SkBF and the cutaneous vascular conductance (CVC) from all possible LDF probe combinations were not statistically different across the three arm and leg trials. Two-way mixed-effects models with absolute agreement were used to compute the intraclass correlation coefficient (ICC) for CVC, and the minimum ICC increased with the addition of LDF probes. The ICC of the average CVC from seven LDF probes was 0.96 between the arm trials and 0.91 between the leg trials, which suggests that there is excellent reliability and little difference between trials following the removal and replacement of the device. Moreover, all individual ICC values from ≥3 LDF probe combinations were greater than 0.70 (i.e., good reliability). These data suggest that SkBF measurements with multiple laser Doppler probes in a custom-made holder have excellent reproducibility after replacing the probes within the same participant. Therefore, this application could provide more reproducible assessments between repeated measurements (e.g., before and after exercise or clinical procedures) where the LDF probes must be removed and replaced within the same location.

## Introduction

Laser light has been frequently used in various biomedical applications since its initial development in the 1960s (Maiman, 1960; Townes, 2007). For decades, researchers have used laser light scattering, reflection, and absorption principles and properties to quantify various physiologic parameters. The optical technique of laser-Doppler flowmetry (LDF) utilizes frequency-shifted laser light signals, which are reflected from red blood cells (erythrocytes), to quantify an index of microcirculatory perfusion in a static tissue (Holloway and Watkins, 1977; Nilsson et al., 1980a; Nilsson et al., 1980b). In human and animal experiments, these LDF probes may be applied to the skin to obtain measurements of skin blood flux (SkBF). The assessment of SkBF *via* LDF probes has provided critically useful information to further our understanding of thermoregulation and microvascular function/dysfunction. However, the LDF technique is not free from limitations, and the assessment of SkBF has been continuously criticized. Namely, it is highly susceptible to motion artifacts, and there is considerably large site-to-site and day-to-day signal heterogeneity. Thus, the inability to remove and replace the LDF probe significantly hinders the ability to obtain observations pre- and post-intervention without the use of normalization techniques (e.g., local vascular occlusion for the prepose of establishing a physiological zero or converting the raw data to a percent change from a thermoneutral baseline (33–34°C), or converting the raw data to a percentage of “maximal” SkBF at the site). Therefore, a more broadly applied method that could achieve greater reproducibility between measurements would benefit physiology researchers.

The LDF measurement is based on the Doppler principle, where laser light is applied to an otherwise static tissue at a specific wavelength (i.e., monochromatic). Due to the light-scattering and absorption properties from endogenous chromophores (e.g., erythrocytes) and other tissue components, reflected laser light becomes frequency-shifted by moving erythrocytes but remains relatively unshifted as it passes through static tissue. The broadening of the laser light, dependent on the velocity of the moving erythrocytes, is subsequently detected by photodetectors and processed by a computer. To date, LDF probes have been applied to various vascularized tissue beds (e.g., skin, muscle, liver, brain, bone, and tooth pulp) in both experiments in humans (Davis et al., 2006; Davis et al., 2008; Ghouth et al., 2018) and animals (Arvidsson et al., 1988; Lombard and Roman, 1990; Hanne et al., 2019; Allen et al., 2020). The exact sampling depth of an LDF probe is difficult to determine. It requires the identification of the individual blood vessel and erythrocyte that has interacted with the light from the LDF probe, which is both site, velocity, and time-dependent. Moreover, increased concentrations of hemoglobin molecules can shield deeper hemoglobin molecules from irradiation. Thus, the strongest signal originates from the erythrocytes closest to the light source, and the signal strength weakens with greater penetration depths. Therefore, it is generally expected that LDF penetration is shallower in tissues with relatively high hemoglobin concentrations, as is the case in skeletal muscle tissue. Whereas in cutaneous measurement, the penetration depth is expected to be slightly deeper. Using a Monte-Carlo model to simulate the diffusion of photons through biological tissue, it is roughly estimated that the sampling depth in cutaneous tissue is between 1.0 and 1.5 mm^2^, yielding a sample volume of roughly ∼1 mm^3^ (Low et al., 2020). Thus, given the anatomical structure of the cutaneous microvasculature, it is generally believed that the LDF signal is generated by arterioles, capillaries, and post-capillary venules of the upper horizontal plexus of the dermis (Braverman et al., 1990). Due to the skin’s large surface area and its superficially located capillary loops (Braverman, 2000), it serves as a readily accessible vascular bed for the investigation of microvascular function (Holowatz et al., 2008).

Similar to LDF measurement, various techniques have been employed to track changes in microvascular perfusion, namely, venous occlusion plethysmography, scanning laser-Doppler imaging, laser-Doppler speckle contrast imaging, and optical coherence tomography (Chaseling et al., 2020; Low et al., 2020). Several other methods have been used to investigate blood flow and vascular function in various regions (e.g., Doppler ultrasonography, flow-mediated dilation, pulse wave velocity, and carotid intima thickness). However, these techniques are primarily isolated to the large conduit arteries and thus, do not fully capture the downstream effects in the microvascular circulation. Other optical techniques such as near-infrared spectroscopy and pulse oximetry provide more detailed information about the interaction between oxygen and hemoglobin. However, due to the high temporal resolution, user independence, and affordability, LDF remains one of the most commonly used tools to monitor SkBF.

Although the skin is generally regarded as an excellent interrogation site for LDF probes, due to its non-invasive accessibility, SkBF measures are incredibly susceptible to motion artifacts and any slight movement or relocation of the LDF probe (Tenland et al., 1983). This is because the skin’s microvascular structure and density are not evenly distributed from site to site (Braverman et al., 1990; Braverman et al., 1992; Braverman and Schechner, 1991). Indeed, several studies have repeatedly demonstrated topographical zones of high and low SkBF using sequential LDF measurements from a localized region (Braverman and Schechner, 1991; Wårdell et al., 1994). Thus, due to the small area measured by a single-point LDF probe, it has been demonstrated that the technique’s reproducibility may be limited as LDF signals vary with slight movements of the probe (Tenland et al., 1983; Braverman et al., 1990; Wårdell et al., 1994; Puissant et al., 2013). Therefore, the LDF measurement is typically used to quantify temporal changes in SkBF in response to pharmacological and thermal stimuli (Low et al., 2020) in experiments where the device is secured in place for the duration of the experiment. Studies utilizing the average resting (i.e., non-normalized) SkBF values as the primary outcome measure or experimental designs where the LDF probe must be removed and replaced (e.g., between-day, pre- and post-exercise, or clinical procedure) have often been considered unreliable. To further improve reproducibility, the influence of spatial inhomogeneities must be reduced. One previous study has used signal averaging techniques of serial measurements in a localized area to improve day-to-day reproducibility (Wårdell et al., 1994). Another group has used an integrated LDF with multiple emitting fibers and two photodetectors showing that this approach improves the coefficient of variation when the device is moved to adjacent positions, 5 mm apart (Salerud and Nilsson, 1986). Based on these prior observations, the present study sought to use a custom-made integrated LDF probe holder (consisting of seven LDF probes) to test the hypothesis that the reproducibility of the LDF signal increases with the addition of individual LDF probes after the device has been removed and replaced within participants in the same location. The development of a method capable of decreasing the spatial inhomogeneities and reproducing measures of SkBF pre- and post-intervention could greatly expand the application of LDF assessments.

## Materials and Methods

### Study Population

Eleven individuals (six men, five women) were invited to participate in the study. The participant characteristics are shown in Table 1. All participants were in good health with no history of cardiovascular, pulmonary, metabolic, or renal disease, and none were taking medications during the study. Each participant had the purposes and risks of the protocol explained to them before written informed consent was obtained. The experimental protocol was approved by the Institutional Review Board of the Penn State Milton S. Hershey Medical Center and conformed with the World Medical Association’s Declaration of Helsinki.

**TABLE 1:** Participant characteristics.

	Value
Sex, *n* (men/women)	6/5
Age, yr	42 ± 15
Height, cm	171.1 ± 7.2
Weight, kg	72.5 ± 8.1
BMI, kg/m^2^	24.7 ± 1.2

Values are means ± SD. BMI, body mass index.

### Measurements

As described in prior reports (Cui et al., 2013; Cui et al., 2021), blood pressure (BP) was measured with an automated sphygmomanometer from the brachial artery (SureSigns VS3, Philips, Philip Medical System). Two sets of laser-Doppler flowmetry systems (MoorLab, Moor Instruments Ltd., Devon, United Kingdom) with a total of seven optical probes were used to measure SkBF. Four of the seven probes were type MP1/7-V2, which had eight glass collecting fibers in a 2 mm ring with one central glass delivery fiber (i.e., a separation distance of 1 mm). Three of the seven probes were type MP12-V2, which had one glass collecting fiber and one glass delivery fiber with 0.5 mm separation of the fibers. Signal penetration depth is multifaceted. It is dependent on the optical properties of the sample tissue and the concentration of erythrocytes in the tissue. Tissues with higher concentrations of erythrocytes will result in a slightly shallower measurement depth. In general, it can be roughly estimated that the LDF signals come from a depth of ∼75% of the separation distance between the delivery and collecting fibers. Thus, the MP1/7-V2 probes are assumed to penetrate slightly further than the MP12-V2 probes. Additionally, the increased number of encircling collecting fibers in the MP1/7-V2 probes will yield a larger sample volume compared to the single-point MP12-V2 probes. All seven probes transmitted light from solid-state laser diodes at ∼780 nm. Each of the probes was calibrated using a calibration kit (CAL, Moor Instruments Ltd., Devon, United Kingdom) before being used in this study.

**FIGURE 1:**
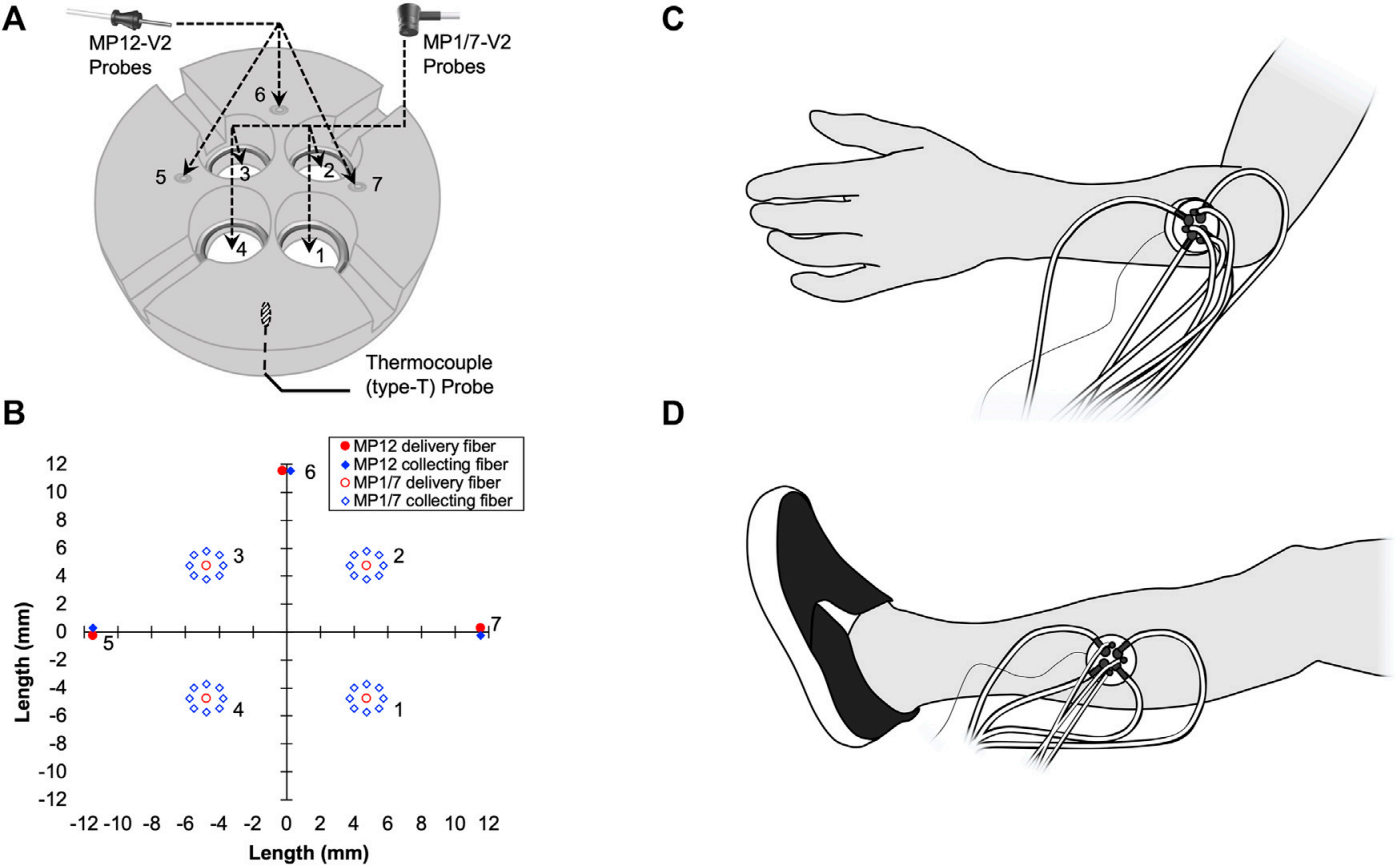
A schematic representation of the custom-made laser Doppler flowmetry (LDF) probe holder **(A)** is shown. Four MP1/7-V2 right angle probes were placed into tapered holes (1, 2, 3, and 4). Three MP12-V2 blunt needle end delivery probes were inserted into three small holes (5, 6, and 7). A thin thermocouple (type T) used to measure skin temperature (Tsk) was placed between the bottom of the probe holder and the surface of the skin. LDF probe-to-probe separation distances in millimeters are plotted **(B)**. The location of the probe holder during the Arm Trial **(C)** and Leg Trial **(D)** are shown.

A custom-made holder was used to mount the seven probes in a circular and equidistant configuration (Figure 1A). Briefly, the mount was made from a 45 mm plastic disc with a height of 11.5 mm. At 11.25 mm from the center of the circle, three small holes (1.25 mm diameter) were drilled at locations 0°, 90°, and 180°, relative to the unit circle. These three holes housed the three MP12-V2 blunt needle end delivery probes. Next, at 7.0 mm from the center of the circle, four larger tapered holes (10.25 mm down-tapered to 8.05 mm diameter) were drilled at locations 45°, 135°, 225°, and 315°, relative to the unit circle. These four tapered holes were specifically drilled to fit the contort of the MP1/7-V2 right angle probe, which has a 3 mm diameter cable. Thus a 4 mm wide by 5 mm high rectangular notch was cut to allow the cable and the right-angle probe to sit flush with the bottom of the holder. Lastly, three small 1.5 mm notches were made along the outside of the holder and used as visual landmarks while replacing the holder on the participant’s skin. Furthermore, an ink marker was used to mark the skin at the location of each notch. LDF probe-to-probe separation distances were consistent (Figure 1B), and LDF probes were placed no closer than 8.4 mm apart. The probe holder was attached to the skin using double-sided adhesive discs (PAD, Moor Instruments Ltd., Devon, United Kingdom) and hypoallergenic tape (Transpore, 3M, St. Paul, MN, United States). To monitor the local skin temperature (Tsk), a thermocouple (TC-2000 thermocouple meters, Sable systems) attached to the skin was placed directly under the probe holder (Figure 1).

### Experimental Protocols

The procedures were conducted with the participant in the supine position in a room with an ambient temperature of ∼23°C. Three trials were performed with intermissions, during which participants were released from the testing table for 10–15 min. The seven probes were placed in the plastic holder. A thin thermocouple (Type T) was also placed under the probe holder. The probe holder was then placed on the participant’s dorsal forearm, approximately at the middle position between the wrist and elbow along the line between the lateral epicondyle of the humerus and the lateral side of the styloid process of radius (Figure 1C). The position of the probe holder was marked using a semi-permanent marker. After instrumentation, participants were asked to rest quietly during an acclimation period prior to the data collection while all signals of the SkBF, Tsk, and BP were monitored. The acclimation period was at least 5 min or until all signals were stable. Thereafter, SkBF and Tsk were recorded for 3 min, while BP was measured once from the contralateral arm (Arm Trial). Then, the probe holder and all of the probes were removed from the forearm. Thereafter, the probe holder and the probes were placed on the lateral aspect of the leg, which was approximately at the midpoint on the line between the tip of the head of the fibula to the tip of the lateral malleolus (Figure 1D). The position of the probe holder on the leg was also marked using a semi-permanent marker. Then, all signals were monitored for at least 5 min for an acclimation period. Thereafter, SkBF and Tsk were recorded for 3 min, while BP was measured once from the arm (Leg Trial). After the probe holder and all of the probes were removed from the leg, the participant was dis-instrumented and released from the testing table. Participants stayed in the laboratory for 10–15 min before lying back down on the testing table. Trials 2 and 3: The probe holder was carefully replaced on the same positions according to the markers left on the skin. The measurements on the forearm and then leg, as well as the intervals between the trials, were repeated. The order of the Arm Trial and Leg Trial was randomized amongst the participants, while the order was kept in the three trials in each individual. Because the cardiovascular variables (BP, HR, *etc*.) and thermoregulatory variables (e.g., body and skin temperatures) can change from day-to-day, these factors will directly affect SkBF. To decrease the possible influences from these factors, the three trials were performed in one laboratory visit.

### Data Analysis

Data were sampled at 200 Hz *via* a data acquisition system (PowerLab, ADInstruments, Castle Hill, NSW, Australia). The mean values of the 3-min recordings of SkBF and Tsk were used as the measurement for the trial. Cutaneous vascular conductance (CVC) was calculated from the ratio of the SkBF to mean arterial pressure (MAP). The SkBF and CVC from each individual probe were calculated at first. The absolute values of BP, Tsk, SkBF, and CVC were used to examine the effects of the intervention (i.e., replacing the probes), and comparisons between the three trials were examined using repeated measures one-way ANOVA. When appropriate, Tukey *post-hoc* analyses were employed.

In this test-retest reliability study, the intraclass correlation coefficient (ICC) of the CVC was used as the primary index for the reliability of the measurement. In the present study, ICC estimates and their 95% confidence intervals were calculated using SPSS statistical software (Version 27, SPSS Inc.) based on a mean-rating (k = 3), absolute-agreement, two-way mixed-effects model (Shrout and Fleiss, 1979). The calculation of ICC for the CVC from each individual probe were performed at first. To test the effects of multiple probes, the mean values of each combination of the CVC from these probes were calculated (e.g., 35 combinations for each three probes). Then, the ICC for the mean CVC from all possible combinations (i.e., 2–7) of the probes were calculated with ICC values ≥0.70 being considered acceptable for research (Matheson G. J., 2019).

Statistical power analysis for ICC was performed using a publicly accessible add-in (Real Statistics Using Excel. www.real-statistics.com, Charles Zaiontz) for Microsoft Excel (Microsoft Corporation, Redmond, WA, United States).

The five-number summary of the distribution (i.e., minimum, Q1, median, Q3, maximum) of ICC values from the combinations of the probes were calculated and are presented with box and whiskers plots using Sigmaplot software (Version 14, Systat Software Inc.). All values are reported as means ± SD. *p* values of <0.05 were considered statistically significant.

## Results

The mean SkBF, Tsk, CVC, BPs, and HR during the three arm trials and three leg trials are shown in Table 2. MAP decreased along the three arm trials (Trial 1 vs Trial 3, *p* < 0.05) and did not change significantly along the leg trials. HR decreased along the three arm trials and did not change significantly along the leg trials.

**TABLE 2:** Absolute hemodynamic and skin microvascular responses.

	Arm				Leg			
	Trial 1	Trial 2	Trial 3	*p*-value	Trial 1	Trial 2	Trial 3	*p*-value
Systolic arterial pressure, mmHg	119 ± 16	115 ± 17	113 ± 15*	0.002	116 ± 17	113 ± 13	110 ± 14*	0.002
Diastolic arterial pressure, mmHg	75 ± 17	74 ± 14	71 ± 15	0.051	74 ± 14	71 ± 14*	69 ± 14*	0.002
Mean arterial pressure, mmHg	90 ± 15	87 ± 14	84 ± 14*	0.002	87 ± 13	86 ± 13	83 ± 13	0.078
Heart rate, bpm	67 ± 16	65 ± 14	62 ± 14*	0.018	62 ± 17	61 ± 15	62 ± 13	0.782
Tsk, °C	29.6 ± 1.7	29.5 ± 3.1	30 ± 3.1	0.904	31.2 ± 0.7	30.9 ± 1.7	29.6 ± 3.6	0.118

Values are means ± SD. Values represent the 3-min resting period prior to replacing the laser Doppler flowmeter probes. The probes were alternated from the arm to the leg every 3 min, a total of three times (trial 1, 2, and 3). Tsk, skin temperature. *P*, p-value of one-way repeated measures ANOVA; *Post-hoc, p < 0.05 vs Trial 1.

Tsk remained constant across all three arm trials and three leg trials. The averaged SkBF and CVC from all probes did not change significantly along the three arm trials or three leg trials (Table 3). The SkBF and CVC from each individual probe during the three arm trials and three leg trials are shown in Table 3. The SkBF and CVC from each individual probe did not change significantly along the three arm trials or three leg trials.

**TABLE 3:** Individual probe measures of skin blood flux and cutaneous vascular conductance.

	Arm				Leg			
	Trial 1	Trial 2	Trial 3	*p*-value	Trial 1	Trial 2	Trial 3	*p*-value
Mean SkBF, p.u.	25.5 ± 9.6	24.3 ± 9.2	23.2 ± 6.7	0.830	28.1 ± 9	28.1 ± 8.8	26.9 ± 9	0.819
Probe 1, p.u.	22.6 ± 17.1	22.5 ± 20.4	19.2 ± 10.9	0.860	23 ± 13	21.8 ± 12.6	18.8 ± 7.8	0.676
Probe 2, p.u.	16.1 ± 7.6	18 ± 9.8	18.9 ± 12.2	0.799	24.6 ± 13.3	24.1 ± 13.8	17.9 ± 7.3	0.351
Probe 3, p.u.	90.8 ± 43.8	77.9 ± 27.5	70.8 ± 19	0.342	99.9 ± 33.7	96.6 ± 26	99.6 ± 48.8	0.974
Probe 4, p.u.	15.6 ± 3.8	19.7 ± 7.9	17 ± 6.6	0.332	20.2 ± 11.8	21.7 ± 9.7	22.5 ± 12.8	0.887
Probe 5, p.u.	10.2 ± 2.4	9.3 ± 2.5	9 ± 2.2	0.432	8.9 ± 2.3	9 ± 2.2	8.9 ± 2.4	0.990
Probe 6, p.u.	13.9 ± 9.7	14.4 ± 14.3	18.2 ± 19.7	0.766	11.2 ± 6.4	13.4 ± 8.4	9.8 ± 7.6	0.525
Probe 7, p.u.	8.9 ± 5.3	8.4 ± 6.2	9.3 ± 7.1	0.947	9 ± 6.3	10.1 ± 6	10.7 ± 6.6	0.830
Mean CVC, p.u.・mmHg-1	28.7 ± 9.3	28.5 ± 10	27.9 ± 7.1	0.811	32.9 ± 10.9	33.5 ± 12.4	32.6 ± 10.2	0.930
Probe 1, p.u.・mmHg^−1^	24.8 ± 4.8	25.7 ± 6.2	22.7 ± 3.2	0.904	26.5 ± 4.4	25.5 ± 4.3	23.4 ± 3.5	0.858
Probe 2, p.u.・mmHg^−1^	18.3 ± 2.5	20.9 ± 2.9	22.5 ± 3.8	0.636	29 ± 4.8	29.1 ± 5.5	22.1 ± 2.8	0.466
Probe 3, p.u.・mmHg^−1^	102.1 ± 12.6	92.5 ± 10.8	87.1 ± 9.2	0.622	116.5 ± 11.8	115.4 ± 11.4	118.8 ± 14.1	0.981
Probe 4, p.u.・mmHg^−1^	17.9 ± 1.6	22.8 ± 2.4	20.3 ± 2.1	0.250	23.1 ± 3.8	25.3 ± 3.4	26.8 ± 4.1	0.793
Probe 5, p.u.・mmHg^−1^	12 ± 1.4	11.1 ± 1.1	11.1 ± 1.1	0.839	10.7 ± 1.2	10.8 ± 1	11 ± 1.2	0.980
Probe 6, p.u.・mmHg^−1^	15.4 ± 3.1	16.8 ± 4.8	20.2 ± 5.8	0.762	13.2 ± 2.5	15.9 ± 3.1	12.6 ± 3.3	0.706
Probe 7, p.u.・mmHg^−1^	10.3 ± 1.8	9.8 ± 2.1	11.2 ± 2.4	0.903	11 ± 2.6	12.4 ± 2.8	13.4 ± 2.7	0.823

Mean SkBF, the average skin blood flux value from all seven LDF probes from the device; p.u., perfusion units; Mean CVC, the average cutaneous vascular conductance value from all seven LDF probes; Probe 1, 2, 3, ..., 7, are the average from all 11 participants for each individual LDF probes are shown. Values are means ± SD. *P*, p-value of one-way repeated measures ANOVA.

**FIGURE 2:**
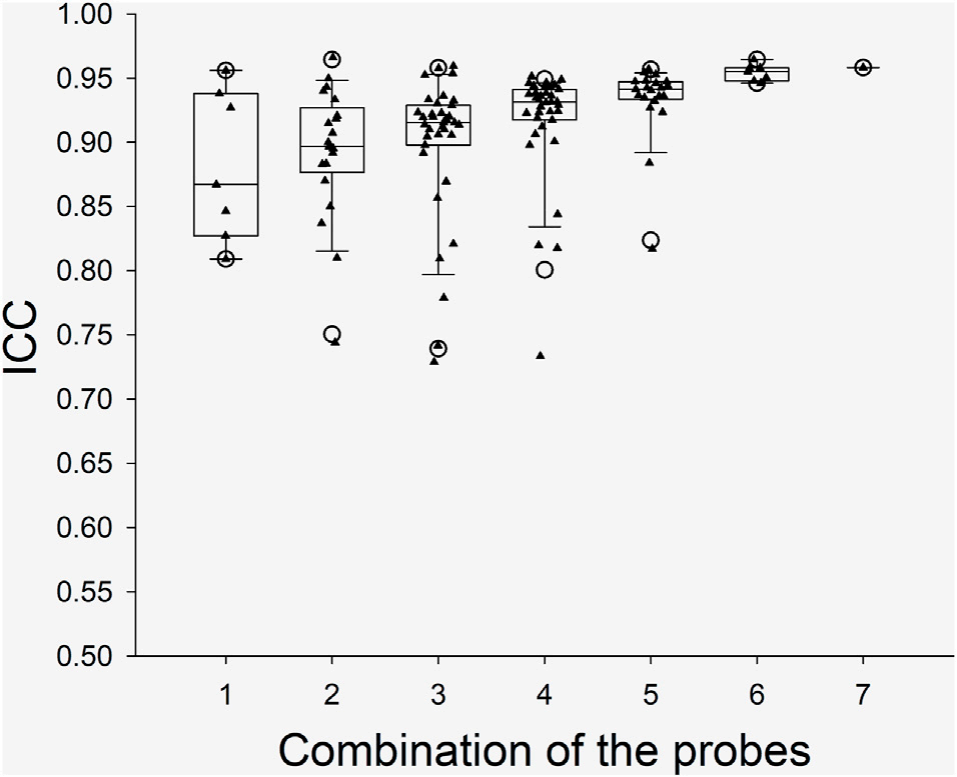
Boxplots for the ICC between CVC values during the Arm Trials with the various probe combinations. *X* axis: the number of probes for the combinations. The calculated “Minimum” and “Maximum” values are indicated by open circles. The data below Minimum or above Maximum were outliers.

**FIGURE 3:**
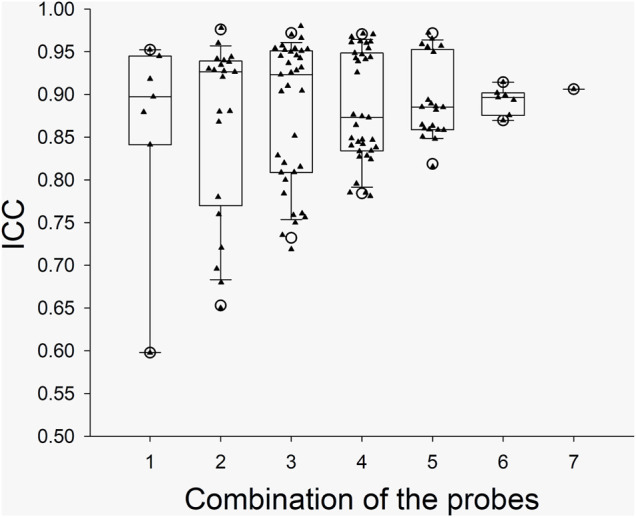
Boxplots for the ICC between CVC values during the Leg Trials with the various probe combinations. *X* axis: the number of probes for the combinations. The calculated “Minimum” and “Maximum” values are indicated by open circles. The data below Minimum or above Maximum were outliers.

The ICC values from the individual probes and the ICC values from the combinations of the probes in the arm trials and in the leg trials are presented with box and whiskers plots in Figure 2 and Figure 3, respectively. The five-number summary of the distribution (i.e., minimum, Q1, median, Q3, maximum) of ICC values in the box and whiskers plots are shown in Table 4. The minimum ICC values (i.e., the lowest detected ICC), generally improved with the addition of probes in both the arm, and leg. Specifically, the lowest ICC with a single probe was only 0.598, while the lowest ICC with seven probes was 0.906, which indicates that using more probes improves the reproducibility of the CVC measurements. All data points from the arm trials using five or more probes and the leg trials with four or more probes had an ICC >0.75, which is considered indicative of good reliability between measurements (Bobak et al., 2018).

**TABLE 4:** The five-number summary of the distribution of ICC values.

Probes (*n* = )	Combinations (*n* = )	Min	Q1	Median	Q3	Max
Arm Trial						
1	7	0.809	0.827	0.867	0.938	0.956
2	21	0.751	0.883	0.897	0.921	0.966
3	35	0.740	0.898	0.915	0.929	0.959
4	35	0.801	0.917	0.931	0.941	0.951
5	21	0.825	0.935	0.941	0.947	0.957
6	7	0.946	0.948	0.955	0.958	0.965
7	1	0.958	0.958	0.958	0.958	0.958
Leg Trial						
1	7	0.598	0.841	0.897	0.945	0.952
2	21	0.651	0.780	0.926	0.938	0.978
3	35	0.733	0.809	0.923	0.951	0.980
4	35	0.781	0.834	0.873	0.949	0.971
5	21	0.816	0.859	0.885	0.950	0.972
6	7	0.870	0.876	0.896	0.902	0.914
7	1	0.906	0.906	0.906	0.906	0.906

Data correspond to the five-number summary of the distribution (i.e. minimum, Q1, median, Q3, maximum) of ICC values which have been presented graphicly in the box and whiskers plots shown in Figures 2, 3.

For the ICC for each of the seven single probes in the arm, the mean statistical power was 0.993 with a range of 0.989–0.997 (*n* = 7). For the ICC for each of the seven single probes in leg, the mean statistical power was 0.993 with a range of 0.990–0.994 (*n* = 7). For the averaged measurements of seven probes, the mean statistical powers were 0.991 for the arm measurements and 0.994 for the leg measurements.

## Discussion

The present study aimed to assess the test-retest reliability of SkBF/CVC measurements with multiple LDF probes in one holder. We found that test-retest reliability of CVC measurements increased with the addition of up to seven LDF probes in an integrated probe holder, which verified our hypothesis. These data suggest that SkBF measurements obtained with multiple LDF probes in a localized area have high reliability after removing and replacing the probes on the same participants. To our knowledge, this is the first study to provide evidence that the absolute values of SkBF/CVC after replacing the probes in the same participants can be used for analysis without the use of a normalization technique.

### Integrated Laser Dopper Flux Probe Design

As illustrated in Figures 1A,B, the integrated LDF probe holder increases total sample volume by ∼ seven-fold. The increase in sample volume is important, as previous studies have shown that spatial inhomogeneities in microvascular density very in relatively small areas in the skin (Braverman et al., 1990; Braverman et al., 1992; Braverman, 1997, Braverman and Schechner, 1991). Thus, the contribution of spatial inhomogeneities should be reduced by simple averaging of the SkBF signals from each LDF probe. Two different types of LDF probes were used in this study to increase the total sample volume. The rationale for using a mixture of probes was related to available space within the custom probe holder, which was cut from a 45 mm diameter acrylic rod. With the addition of three smaller-diameter MP12-V2 probes, the probe-to-probe separation distance was reduced while increasing the number of probes from four to seven. The present data show that by this addition of probes within the same general area, signal reliability improves (Figures 2, 3). However, it is important to note that the sampling depth between the two types of probes is slightly different. Although, determining the exact penetration depth requires the identification of the individual blood vessel and red blood cell that has interacted with the light from the LDF probe(s), which is both site, perfusion, and time-dependent. Thus, it is impossible to know the exact penetration depth. However, using Monte-Carlo modeling techniques for the diffusion of photons, it is estimated that the sampling depth in cutaneous tissue is 1.0–1.5 mm (Low et al., 2020). It is also speculated that that more superficial hemoglobin molecules could shield deeper hemoglobin molecules from irradiation. Thus, the strongest signal could likely originate from the superficial dermis vasculature (i.e., erythrocytes more superficial in relation to the device), and the signal strength weakens with greater penetration depths. In the present study, the data suggest that only four of the deeper-penetrating MP1/7-V2 probes are sufficient. As indicated by the majority of ICC values >0.75, which is considered indicative of good reliability between measurements (Bobak et al., 2018). However, the addition of the three MP12-V2 probes did indeed further improve reliability. Another potential issue is the physical displacement of the device following intermission periods between trials. The LDF probes were secured within the probe holder during measurement periods and were not moved within the probe holder itself. Additionally, an ink marker was used to outline the device and used to identify the probe location. Indeed, the present study did not appear to produce any inter-operator variability as the device was assembled, removed, and replaced by several members of the study team.

With the current integrated probe holder design, it is important to consider the possibility of probe-to-probe light contamination with the custom probe holder. We placed the probes in a configuration where probe light sources were no closer than 8.4 mm (Figure 1B). We selected these probe distances as they did not produce any detectible change in the absolute SkBF signal whilst the probes were individually turned on and off repeatedly. Thus, we believe that the LDF probes were placed at a sufficient separation distance to avoid probe-to-probe light contamination. Although, it must be stated that while we did not observe any change in the SkBF signals using these probe-to-probe separation distances, closer separation distances than 8.4 mm were not explicitly tested. Therefore, it remains unclear if closer separation distances produce noticeable changes in the LDF signal that can be attributed to light contamination from an adjacent probe. Studies by Braverman and collogues showed the randomly dispersed ascending arterioles, which supply the horizontal vascular network of capillaries, were at 1.5–1.7 mm intervals in the derma (Braverman et al., 1990). Thus, we believe that the ascending arterioles within the sample volumes of each of the seven individual LDF probes were most likely different.

### Laser Doppler Flux and Cutaneous Vascular Conductance Responses

LDF SkBF value is a relative unit of perfusion (p.u.), commonly termed “red blood cell flux” or simply “flux,” which is defined by the manufacturer using a phantom. Specifically, flux is the product of measured red blood cell concentration and velocity; thus, flux is proportional to the velocity and concentration of erythrocytes in a local tissue and is largely impacted by changes in cutaneous vasoconstriction and dilation. SkBF signals can be isolated to capillaries with a diameter of roughly 10 microns which are located in the papillary dermis 1–2 mm below the epidermal surface (Braverman, 1997). In the present study, no statistical differences were observed between the individual or mean SkBF values (Table 2). Thus, it is reasonable to assume that the addition of probes within the integrated probe holder contributed to the signal stability across trials at the same measurement location within each participant.

Similarly, with the SkBF values, the CVC values were not significantly different between trials in either the arm or leg (Table 2). The CVC was calculated as flux/mean arterial pressure. Obtaining a stable and reproducible CVC value was of particular interest in the present study due to its potential application in future studies. Indeed, in most thermoregulation studies, the main interest is on the cutaneous vascular tone (i.e., vasoconstriction and/or dilation) and less commonly focused on cutaneous blood flow itself. The BP is the main driving force for the blood flow. To decrease the effects of BP in the data, the current investigation reports CVC values, as the calculation is often used to represent the cutaneous vascular tone. In the present study, the BP decreased over time. It can be assumed that this decrease in BP is largely due to the experimental conditions in that all participants were monitored in a quiet room for over 1 h without any strong physical activity or emotional stimuli. As discussed above, the influences of this BP change should be decreased by using CVC. Temperature change (cooling or heating) can directly cause cutaneous vasoconstriction or vasodilation, which will alter CVC. In the present study, there were no changes in the skin temperature at the sites in the three trials (Table 1). Moreover, we could also suppose that other environmental factors such as room temperature and humidity were maintained during the study. Thus, the changes in CVC values in the present study should not be a physiological response to these factors (BP or temperature, *etc*.).

### ICC of Intraindividual and Intra-site Measurements Between Three Trials

This study utilized the assessment of ICC to reflect measurement reliability between trials where the LDF probes were removed and replaced within participants and on the same skin sites (e.g., the forearm and leg). Reliability reflects the extent to which measurements can be replicated in a sample of participants. Mathematically, reliability represents a ratio of true variance over true variance plus error variance (Ebel, 1951). The evaluation of reliability is fundamental to research and clinical assessment because true values can only be estimated. The assessment of ICC during repeated tests is widely used to determine the reliability of a measurement (Carrasco et al., 2014). Although there are several models to calculate ICC, ICC reflects both degree of correlation and agreement between measurements from the same participant (i.e., class). The ICC value ranges between 0 and 1, with values closer to 1 representing stronger reliability. It has been suggested that ICC values less than 0.5 indicate poor reliability, values between 0.5 and 0.75 indicate moderate reliability, values between 0.75 and 0.9 indicate good reliability, and values greater than 0.90 indicate excellent reliability (Koo and Li, 2016).

The readings from LDF probes can be significantly altered when the site is slightly moved (Braverman et al., 1990). Therefore, the reliability of SkBF and CVC measurements with single-point LDF is low, as several prior studies have shown (Roustit et al., 2010). It has been shown previously that when a single-point probe is reapplied to the same skin sites within-participants, the reproducibility is greatly improved (Yvonne-Tee et al., 2005). Likewise, when multiple probes in proximity are averaged, the coefficient of variation is reduced (Salerud and Nilsson, 1986). Thus, we speculate that increasing the total measurement area through the addition of multiple LDF probes would help to buffer the influences from inhomogeneities in the skin microvasculature and provide a more reproducible measure of CVC between measurement trials in the same participants.

In our study, the SkBF (and thus CVC) measurements were recorded from 14 individual sites (i.e., seven in the arm and seven in the leg). The ICC of CVC was calculated from the individual sites and the possible combinations in each trial. To ensure the reliability of the measurement, we think that the minimum value from the box and whiskers plots should be predominately considered. For the data from each single probe/site, the minimum ICC values were ∼0.60 and 0.81 at leg and arm, respectively, which indicate a moderate to good reliability. However, considering that the application of LDF will not be limited to the forearm, the SkBF/CVC measurement with a single probe has only moderate reliability. When multiple probes (>3) were used, the ICC values increased with the number of the probes (Figures 2, 3). We speculate that the relatively reduced ICC for 1 – 2 probes could be due to the measured area being small. With the increase in the number of probes, the total measured area increased, which in turn increased the ICC. Although there was one outlier data point, the Minimum ICC of CVC with four probes was greater than 0.7, which indicates are good to excellent reliability (Cicchetti, 1994).

### Limitations

In the present study, there are several limitations that must be considered when interpreting these data or implementing the proposed approach for physiology research studies. First, the intermission period, which was fixed at 10–15 min, could be initially viewed as a limitation of the study since it is unclear if the measures are reproducible with longer intermissions. However, this period was necessary to establish that any observed changes in skin blood flow, if any, were from the approach itself and not a result of “real” SkBF changes that are known to fluctuate throughout the day. The possible changes in BP, central blood volume, skin and central temperature, and environmental factors such as humidity from days to weeks will actually alter SkBF. In the current study, the SkBF/CVC remained stable between all three trials in both the arm and leg. Thus, it is unlikely that these factors significantly contributed to the measurements. Second, without the use of a normalization procedure, it remains difficult to compare between various sites and individuals using the absolute SkBF and CVC values in human studies. Indeed, a prior study has shown that the absolute resting CVC values within participants were poor between days but improved with the application of heat strain (Gemae et al., 2021). Thus, caution is advised when interpreting SkBF data where comparisons between individuals or locations are made. Third, we acknowledge that the number of the individual subjects was not large (*n* = 11) as those in prior test-retests reliability studies (Koo and Li, 2016). However, the data of the measured CVC in the three trials were quite stable, which led to high ICCs. Importantly, the ICC power calculations for both the arm and leg trials in both individual and averaged CVC conditions were sufficiently high. Thus, the total sample size in the present study was sufficient. Fourth, given the present sample size for males (*n* = 6) and females (*n* = 5) or for age range (*n* = 6 for <40 years old, *n* = 5 for >40 years old), the current study was unable to compare between biological sex or various age groups. Thus, this highlights a significant area for future investigations with more subjects. Lastly, as is the case in many physiology experiments, there may be a potential limitation of the measurement device itself as manufacturing discrepancies cause variation in the absolute values from probe to probe. It cannot be overlooked that in the present study, LDF probe 3, on average, provided higher readings across all participants than that of the other probes. This is likely due to slight differences in the manufacturing of each probe. Although each probe was calibrated using the manufacturer’s calibration procedure, there may still be discrepancies between probes and connections to the light source, which is housed in a separate device. Cupping connectors that join each fiberoptic cable may also have slight variations contributing to the variable readings. However, as these readings were reproducible across all trials (i.e., there were no significant changes in the absolute values between trials), the relatively high reading from probe three did not appear to detract from the original findings of this study.

### Application of Integrated Laser Doppler Flux Probes in Research

The current study shows the addition of ≥4 LDF probes in an integrated probe holder device is sufficient for physiology research in humans. Prior to the development of this approach, our laboratory and others had been significantly limited by the LDF measurement in experiments where it is not realistic to measure SkBF continuously. Until now, protocols where the lasers had to be physically removed from the participant to complete other interventions significantly confounded the comparison of the SkBF measurement. With this new approach, various experimental designs, previously confounded by the limitation of a relatively small sample volume, may be better reliably investigated. For instance, this approach can be used in the protocols using whole-body heating with water immersion, acute exercise, or exercise training over a period of days or weeks. Moreover, the data from the multiple probes could present an “averaged” condition, effectively minimizing the effects of skin inhomogeneities. In turn, the study measuring reactive changes in blood flow, we speculate that the proposed application may also accurately track “averaged” reactive changes in SkBF and CVC. Importantly, the ability to remove and replace the LDF device allows for greater application from research in the clinical setting. As patients are routinely transferred between locations for certain diagnostic tests and procedures, the proposed approach could allow for a more reliable pre- and post-assessment of SkBF and CVC. Moreover, it is speculated that this approach could be applied to monitor the skin microcirculatory function in certain disease progressions (e.g., diabetes or peripheral artery disease) within the patients, as well as track the effects of medical therapy or surgical interventions (i.e., revascularization or skin graft) over time. It is also reasonable to speculate that this approach could be applied to animal research as well. In animal models, more invasive techniques allow for the assessment of flux from various tissues, including but not limited to the skeletal muscle. However, due to the size of the animal model, a different arrangement of probes and thus a revised integrated probe holder may be necessary to facilitate a smaller sampling area. Moreover, considering that the total sizes of the organs (e.g., skin in one limb) in small animals (e.g., mice or rats) are small, the relative area measured by multiply LDF probes can be relatively much larger (e.g., over 30%). Under this condition, it is possible that measurements could be used for inter-individual and inter-site comparisons, while further studies are needed to confirm this. Thus, the approach may have even greater applicability in these experiments as well.

In conclusion, the present study showed that increasing the number of individual LDF probes in a custom-made holder increases the reliability of the LDF measurement during test retests. These results suggest that CVC measurements, obtained using multiple laser Doppler probes (number ≥4), have a sufficiently high degree of reproducibility after replacing the probes within the same participant. Thus, we suggest the values from multiple laser Doppler probes in a holder can be used in the intraindividual analysis for certain experiments where the LDF probes must be removed and replaced in the same participants (Anderson and Parrish, 1981).

## Data Availability

The raw data supporting the conclusions of this article will be made available by the authors, without undue reservation.

## Abbreviations

BP, blood pressure; CVC, cutaneous vascular conductance; HR, heart rate; ICC, intraclass correlation coefficient; LDF, laser Doppler flowmetry; MAP, mean arterial blood pressure; Q1, first quartile; Q3, third quartile; SkBF, Skin blood flow; Tsk, local skin temperature.
